# Ultrasonographic cervical length screening at 20–24 weeks of gestation in twin pregnancies for prediction of spontaneous preterm birth: A 10-year Taiwanese cohort

**DOI:** 10.1371/journal.pone.0292533

**Published:** 2023-10-05

**Authors:** Ksenia Olisova, Chih-Hsuan Sao, Eric C. Lussier, Chan-Yu Sung, Peng-Hui Wang, Chang-Ching Yeh, Tung-Yao Chang

**Affiliations:** 1 Department of Medical Research, Taiji Clinic, Taipei, Taiwan; 2 Department of Obstetrics and Gynecology, Taipei Tzu Chi Hospital, Taipei, Taiwan; 3 Institute of Clinical Medicine, National Yang Ming Chiao Tung University, Taipei, Taiwan; 4 Department of Obstetrics and Gynecology, Taipei Veterans General Hospital, Taipei, Taiwan; 5 Department of Obstetrics and Gynecology, School of Medicine, National Yang Ming Chiao Tung University, Taipei, Taiwan; 6 Female Cancer Foundation, Taipei, Taiwan; 7 Department of Medical Research, China Medical University Hospital, Taichung, Taiwan; 8 Department of Fetal Medicine, Taiji Clinic, Taipei, Taiwan; McGill University, CANADA

## Abstract

**Background:**

Shortened cervical length is one of the primary predictors for spontaneous preterm deliveries in twin pregnancies. However, there is lack of consensus regarding cut-off values. Recent evidence highlights that established cut-offs for cervical length screening might not always apply across different populations. This study aims to present the distribution of cervical length in Taiwanese twin pregnancies and to assess its predictive value for spontaneous preterm birth during mid-trimester screening.

**Materials and methods:**

This retrospective analysis of cervical length screening in Taiwan evaluated 469 twin pregnancies between 20–24 weeks of gestation. Outcome data were obtained directly from the medical records of the delivery hospital. The study explored the predictive value of cervical length screening for spontaneous preterm birth and the characteristics of cervical length distribution in Taiwanese twin pregnancies.

**Results:**

The average gestational age at screening was 22.7 weeks. Cervical length values displayed a non-normal distribution (*p-value* <0.001). The median, 5^th^ and 95^th^ centiles were 37.5 mm 25.1 mm, and 47.9 mm, respectively. Various cut-off values were assessed using different methods, yielding positive [negative] likelihood ratios for spontaneous preterm births between 32–37 weeks of gestational age (GA) (1.3–30.1 and [0.51–0.92]) and for very preterm births between 28–32 weeks GA (5.6–51.1 and [0.45–0.64]).

**Conclusions:**

The findings from our analysis of Taiwanese twin pregnancies uphold the moderate predictive potential of cervical length screening, consistent with prior investigations. The presented likelihood ratios for predicting preterm birth at different gestational ages equip clinicians with valuable tools to enhance their diagnostic rationale and resource utilization. By fine-tuning screening parameters according to the spontaneous preterm birth prevalence and clinical priorities of the particular population, healthcare providers can enhance patient care. Our data implies that a cervical length below 20 mm might provide an optimal balance between minimizing false negatives and managing false positives when predicting spontaneous preterm birth.

## Introduction

Despite advancements in medical care, the incidence of preterm delivery (5% to 18%) has not decreased in recent years [1, 2]. Preterm birth (PTB) is known to be associated with high rates of perinatal mortality and morbidity [3], increasing financial burden on health care systems. Twin pregnancies have a PTB rate almost 10 times higher than singletons, with PTB incidence in twins reaching as high as 60% [2–4].

In singleton pregnant women whose cervices were classified as short at mid-trimester cervical length (CL) screening, risks of spontaneous PTB (sPTB) have shown to increase [5–7]. Goldenberg et al. screened 147 twin pregnancies and found that a cervical length of ≤ 25 mm at 24 weeks of gestation was the best predictor of sPTB [8]. Over the past 10 years, the association between cervical length in the second trimester and sPTB for twin pregnancies has been described [9–11]. Researchers have proposed various CL cut-offs for identifying high-risk twin pregnancies: 20 mm [12], 22 mm [13], 25 mm [14], 30 mm [15], and 36 mm [16]. Despite the extensive research on the relationship between CL and sPTB, the best cut-off value for predicting spontaneous preterm birth still lacks consensus. Sisti [17] underlined an ongoing debate over whether using ROC curve analysis or the 5^th^ centile is the best way to determine the optimal cut-off value.

Current screening protocols for predicting sPTB in twin pregnancies are inconsistent in definitions of sPTB, cut-off values of short cervical length, and gestational age (GA) at screening [18–23], resulting in varying outcomes. Besides, most studies on cervical length distribution were conducted in Caucasian women [21, 24]. Additional evidence shows that cervical length might differ depending on race or ethnicity [25, 26]. Also, likelihood ratios for different conditions are in desperate need for the improvement of diagnostic reasoning [27, 28]. The main objectives of this study were to provide data regarding the distribution of cervical length in twin pregnancies and to evaluate the predictive value of second-trimester cervical length screening in Taiwan.

## Materials and methods

This retrospective cohort study of mid-trimester ultrasound screening in twin pregnancies at a private specialized fetal medicine clinic was conducted between November 2008 and December 2018. Cases were either referred by local obstetricians or self-referred. Medical records were reviewed for information on maternal age, parity, GA at presentation, conception method, and chorionicity.

Cervical length was measured transvaginally and recorded in millimeters (mm) by experienced sonographers at 20–24 weeks GA following guidelines provided by the International Society of Ultrasound in Obstetrics and Gynecology (ISUOG) [29]. Subsequent pregnancy management was at the referring obstetricians’ discretion. Maternal complications and neonatal outcomes were reviewed from the medical records of the delivery hospital. All methods were performed under relevant guidelines and regulations. Given the study’s retrospective nature, research approval and a waiver of patient informed consent were granted by the ethics committee from Taipei Veterans General Hospital (IRB# 2020-02-008BC) under the premise of ensuring patient confidentiality.

We used sPTB at <34 weeks’ gestation as a primary outcome, and sPTB at <28, <32, <37 weeks as secondary outcomes. The sPTB was defined as delivery after preterm premature rupture of membranes or as spontaneous onset of preterm labor (shortening and dilation of the cervix with uterine contractions) [30]. Inclusion criteria were women with viable twin pregnancies who visited our clinic for a mid-trimester anatomical ultrasound examination and received a transvaginal CL measurement between November 2008 and June 2018. Owing to retrospective observational design, the type of delivery (spontaneous/iatrogenic) was known in our sample, as a result, cases who had iatrogenic delivery at < 37 weeks were excluded from the final analysis to avoid over- or underestimation of predictive value. We excluded cases of twin-to-twin transfusion syndrome, monochorionic monoamniotic pregnancies and cases with a cerclage placed before the screening. Patients with incomplete pregnancy data were also excluded. Count (*n*), percentage (*%*), mean ($\bar{x}$), and standard deviation (*SD*) were reported to describe the study sample. Stratified analysis by delivery before or after 34 weeks of gestation was conducted by Chi-square (*X*^*2*^) or t-test, the same tests were used for supplementary stratified analysis by different interventions. Cervical length distribution was checked for normality using the Kolmogorov-Smirnov test. The alpha significance level was set *a priori* for two-tailed p-value at <0.05. We employed univariate logistic regression to assess the relationship between sPTB at GA<34 weeks and cervical length at 20–24 weeks of gestation. Due to the limitations of retrospective study design and inclusion of the cases who underwent preventative and therapeutic measures (progesterone, cerclage, tocolytics), we also conducted multivariable logistic regression adjusting for possible confounding factors, including the above mentioned interventions, maternal characteristics (age, parity, conception method), and current pregnancy characteristics (gestational diabetes, preeclampsia, PPROM, chorionicity, mode of delivery, presence of funneling). The receiver operating characteristics (ROC) curve was used to evaluate the diagnostic ability of cervical length to predict sPTB at GA <37, <34, <32, and <28 weeks. To provide data on predictive value, we reported sensitivity, specificity, positive/negative predictive value (PPV/NPV), positive/negative likelihood ratio (LR+/LR-), false positive rate (FPR≈*1-Specificity*), false negative rate (FNR≈*1-NPV*) and accuracy for chosen cut-offs (based on 5^th^, 10^th^ centile, Youden’s index = (*Sensitivity+Specificity-1*), and F1-score = ($\frac{2\times Precision\times Recall}{Precision+Recall}$) values) to predict sPTB at < 28, 32, 34, and 37 weeks of gestation. Youden’s index and F1-score values range between 0 and 1, where Youden’s index = 1 suggests that the test produced no false positives or false negatives, and F1-score = 1 indicates perfect precision and recall. The likelihood ratios imply how much a test result will raise or lower the pretest probability. While likelihood ratio of 1 suggests that the posttest probability equals to pretest probability, LR+ above 1 suggests an increase in posttest probability and LR- below 1 suggests a decrease in posttest probability. The greater the LR+ and the smaller the LR-, the more apparent is the change in posttest probability [31]. Data were analyzed by R (version 1.2.5033, Rstudio Inc.).

## Results

### Study sample characteristics

In total, 469 twin pregnancies met the inclusion criteria for this retrospective cohort study (Table 1). The mean maternal age was 34 years, with approximately one-third of the participants being over 35 years old. Nulliparous women accounted for 75.3% of the sample, and 70.1% of the pregnancies were conceived using assisted reproductive technologies (ART), including 11.9% by intrauterine insemination (IUI) and 58.2% by in vitro fertilization (IVF). Most pregnancies (85.7%) were dichorionic diamniotic (DCDA) twins, while 14.3% were monochorionic diamniotic (MCDA) twins. Progesterone treatment was received by 40% of the women during pregnancy (as a part of ART protocol or as a preventative measure for sPTB), and tocolytics were administered to approximately 33% of the participants. PPROM occurred in 22.6% of cases. A total of 164 women (35%) delivered prematurely, with 67.1% of those sPTBs occurring between 34 and 37 weeks of gestation. Cesarean section was the most common mode of delivery, accounting for 90.4% of cases. The stratified analysis by delivery before or after 34 weeks of gestation showed a significant difference (p-value = 0.009) in the rate of sPTB at <34 weeks for dichorionic and monochorionic pregnancies. Mean CL significantly differed between groups (p-value < 0.001), with measurements of 38.1 ± 6.3 mm for those born >34 weeks and 29.6 ± 13 mm for those born preterm. The presence of funneling was also associated with higher rates of sPTB at <34 weeks (p-value < 0.001). Cases requiring interventions in the form of tocolysis (p-value < 0.001) or cerclage (p-value = 0.009) had higher rates of sPTB. Nearly 65% (n = 106) of the study sample who delivered prematurely experienced PPROM.

**Table 1:** Descriptive statistics of the study sample (n = 469) and comparison between those who had spontaneous delivery before and after 34 weeks of gestation.

Characteristic	Total, n = 469, $\bar{x}$ ± SD/n (%)	Gestational age at delivery >34, n = 415, $\bar{x}$ ± SD/n (%)	Gestational age at delivery <34, n = 54, $\bar{x}$ ± SD/n (%)	*p-value* ^i^
**Maternal Age (years)**	34.2 ± 3.8	34.3 ± 3.8	33.8 ± 3.7	0.39
**Maternal Age > 35 years**	163 (34.8%)	228 (34.9%)	18 (33.3%)	0.82
**Gestational diabetes**	18 (3.8%)	14 (3.4%)	4 (7.4%)	0.15
**Preeclampsia**	19 (4.1%)	17 (4.1%)	2 (3.7%)	0.89
**Nulliparity**	353 (75.3%)	313 (75.4%)	40 (74.1%)	0.83
**Conception**				0.91
** Spontaneous**	140 (29.9%)	123 (29.6%)	17 (31.5%)	
** ART** ^**a**^ **(IVF** ^**b**^ **+IUI** ^**c**^ **)**	392 (70.1%)	292 (70.4%)	37 (68.5%)	
** IVF**	273 (58.2%)	243 (58.6%)	30 (55.6%)	
** IUI**	56 (11.9%)	49 (11.8%)	7 (13%)	
**Chorionicity**				0.01**
** DCDA** ^**d**^	402 (85.7%)	362 (87.2%)	40 (74.1%)	
** MCDA** ^**e**^	67 (14.3%)	53 (12.8%)	14 (25.9%)	
**Cervical length (mm)**	37.1 ± 7.8	38,1 ± 6.3	29.6 ± 13.0	<0.001***
**PPROM** ^**f**^	106 (22.6%)	68 (16.4%)	38 (70.4%)	<0.001***
**Funneling**	12 (2.6%)	2 (0.5%)	10 (18.5%)	<0.001***
**Intervention**				
** Progesterone**	187 (39.9%)	163 (39.3%)	24 (44.4%)	0.47
** Tocolysis**	155 (33.0%)	113 (27.2%)	42 (77.8%)	<0.001***
** Cerclage**	7 (1.5%)	4 (1.0%)	3 (5.6%)	0.01**
**Delivery mode**				0.56
** Vaginal**	45 (9.6%)	41 (9.9%)	4 (7.4%)	
** Cesarian section**	424 (90.4%)	374 (90.1%)	50 (92.6%)	
**Cervical length <20mm**	16 (3.4%)	4 (1%)	12 (22.2%)	<0.001***
**GAD**^**g**^ **(weeks)**	36.3 ± 2.4	37.0 ± 1.1	30.9 ± 2.6	<0.001***
**IUGR** ^**h**^	12 (2.6%)	11 (2.7%)	1 (1.9%)	0.73

^a^ Assisted reproductive technology

^b^ In vitro fertilization

^c^ Intrauterine insemination

^d^ Dichorionic diamniotic

^e^ Monochorionic diamniotic

^f^ Preterm premature rupture of the membranes

^g^ Gestational age at delivery

^h^ Intrauterine growth restriction

^I^ Chi-square test (X^2^) for categorical or t-test for continuous variables

* p-value ≤0.05

** p-value ≤0.01

*** p-value ≤0.001.

### Cervical length distribution

**Fig 1:**
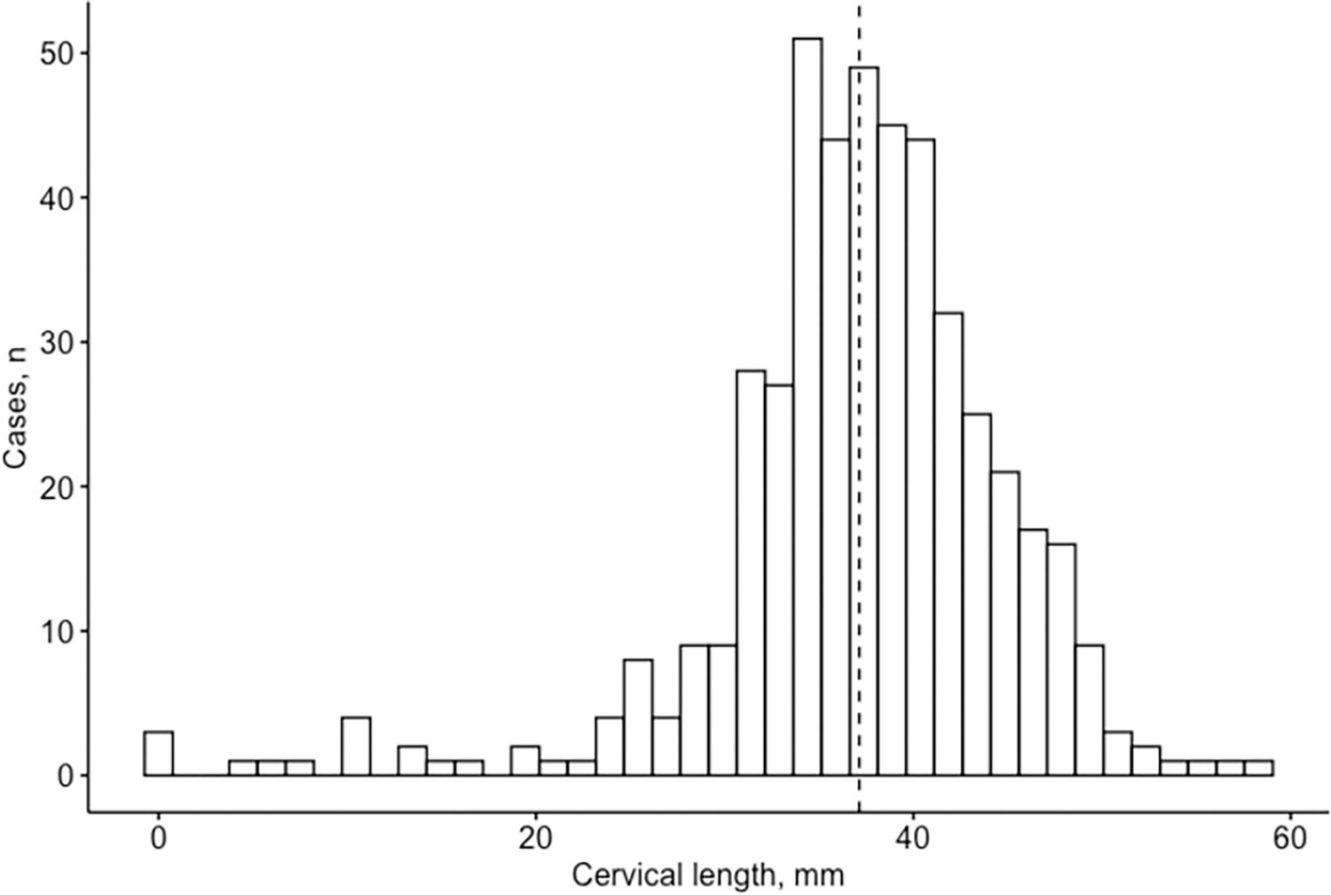
Distribution of cervical length in the study group at 20–24 weeks of gestation. Median = 37.5 mm (dashed line).

The distribution of the cervical length at 20–24 weeks of gestation had a mean of 37.1 mm and a standard deviation of 7.8 mm (Fig 1). According to the Kolmogorov-Smirnov test (p-value <0.001), cervical length measurements did not follow a normal distribution. The distribution exhibited left skewness (-1.41, p-value<0.001) and kurtosis of 4.8. The median cervical length measurement was 37.5 mm, ranging from 0 to 58.3 mm. The mean gestational age at transvaginal cervical length screening was 22^+5^ weeks. The 5^th^ and 95^th^ centiles were 25.1 mm and 47.9 mm, respectively (Table 2).

**Table 2:** Commonly used percentiles for cervical length (mm).

Percentile	1^st^	2.5^th^	3.4^th^	5^th^	10^th^	25^th^	50^th^	75^th^	90^th^	95^th^	97.5^th^	99^th^
**Cervical length (mm)**	6.9	14.5	20.0	25.1	30.4	33.9	37.1	41.7	46.1	47.9	49.3	51.9

### Association between cervical length and rate of spontaneous preterm birth

Univariate logistic regression analysis (Table 3) revealed that each 1 mm increase in cervical length measurement was associated with an 11% decrease in the odds of spontaneous preterm birth at <34 weeks of gestation (Model 1, OR = 0.89, 95% CI: 0.86–0.92, p-value < 0.001). This association remained significant even after adjusting for possible confounding factors, such as interventions (Model 2), maternal characteristics (Model 3), and current pregnancy characteristics (Model 4, adjusted odds ratio = 0.92, 95% CI: 0.87–0.97, p-value = 0.001).

**Table 3:** Results of univariate and multivariable logistic regression models for prediction of spontaneous preterm birth before 34 weeks in twin gestation (n = 469).

	Covariates	Model 1, OR (95% CI)	Model 2, aOR (95% CI)	Model 3, aOR (95% CI)	Model 4, aOR (95% CI)
	**CL, mm**	0.89 (0.86;0.92)***	0.90 (0.86;0.94)***	0.90 (0.86;0.94)***	0.92 (0.87;0.97)**
**Interventions**	**Progesterone**	-	0.82 (0.41;1.59)	0.86 (0.42;1.74)	0.98 (0.47;2.05)
	**Tocolysis**	-	7.96 (4.00;16.86)***	8.04 (4.02;17.15)***	7.70 (3.74;16.99)***
	**Cerclage**	-	0.31 (0.03;2.38)	0.30 (0.03;2.33)	0.07 (0.01;1.27)
**Maternal characteristics**	**Maternal age**	-	-	0.99 (0.91;1.09)	1.00 (0.91;1.09)
	**Parity**	-	-	1.01 (0.52;1.85)	0.94 (0.46;1.75)
	**Conception:**	-	-		
	**Spontaneous**	-	-	Ref.	Ref.
	**IUI**			1.11 (0.33;3.39)	1.76 (0.43;6.91)
	**IVF**	-	-	0.75 (0.35;1.67)	1.27 (0.48;3.71)
**Pregnancy characteristics**	**GDM**	-	-	-	1.93 (0.47;6.58)
	**PE**	-	-	-	0.88 (0.12;3.79)
	**Chorionicity:**	-	-	-	
	**DCDA**	-	-	-	Ref.
	**MCDA**	-	-	-	3.49 (1.20;10.55)*
	**Delivery mode:**				
	**Vaginal**	-	-	-	Ref.
	**Cesarian section**	-	-	-	1.62 (0.50;6.79)
	**Funneling**	-	-	-	12.79 (1.24;318.69)

^a^ Odds ratio

^b^ Confidence interval

^c^ Adjusted odds ratio; Univariate and multivariable logistic regression

*** p-value <0.001

** p-value < 0.01

* p-value<0.05.

**Fig 2:**
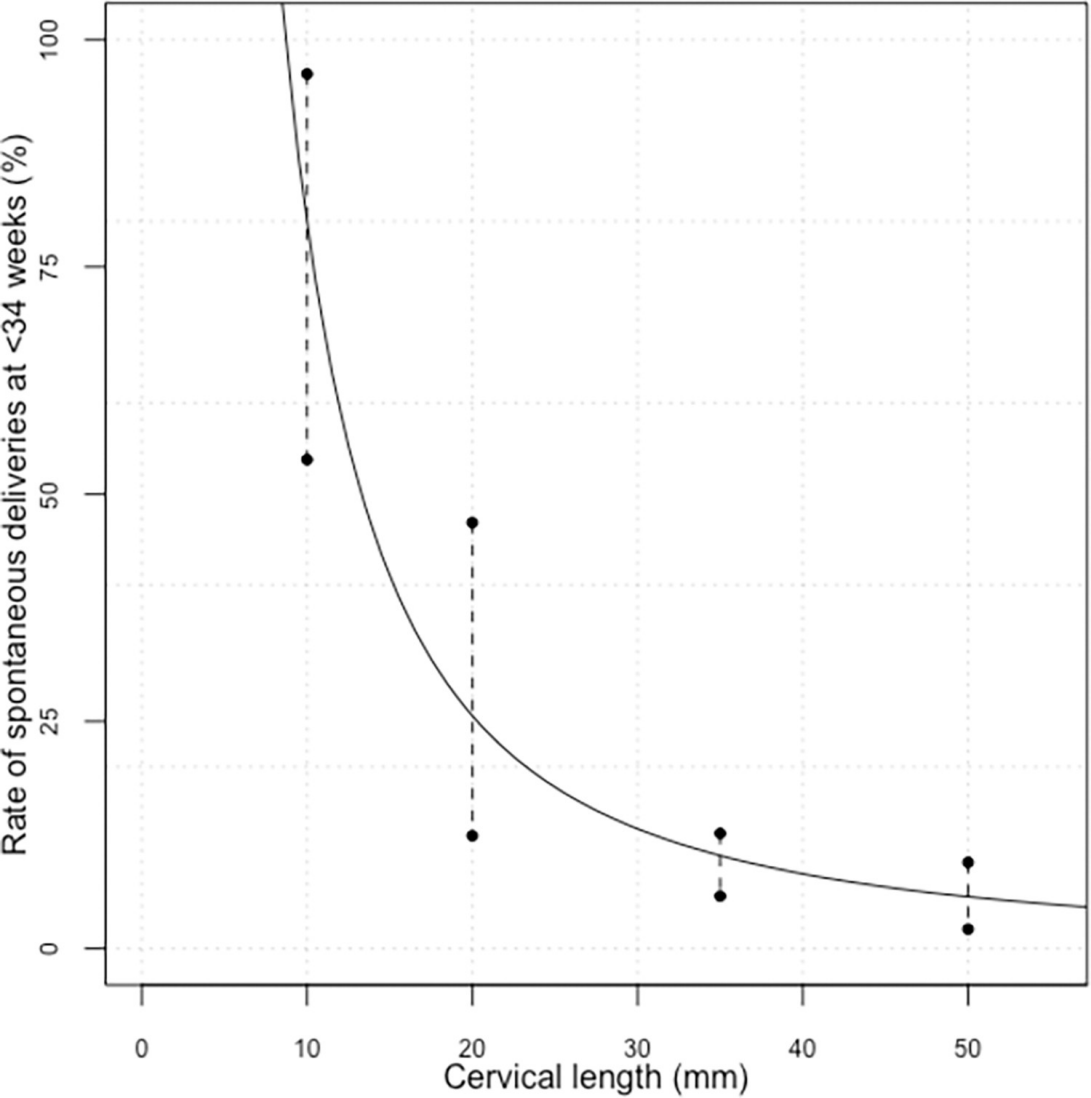
Rate of spontaneous deliveries before 34 weeks and 95% confidence intervals according to cervical lengths measured at 20–24 weeks of gestation. 95% Confidence interval (Dashed line with round end cap).

Fig 2 visually depicts an inverse relationship between measured cervical length and the risk of spontaneous delivery prior to 34^th^ week of gestation. Notably, those women with cervical length measurements above 40 mm had a preterm birth rate about 6%. Conversely, those with cervical length measurements below 10 mm had a preterm birth rate of approximately 75%.

### Predictive value of cervical length screening

**Fig 3:**
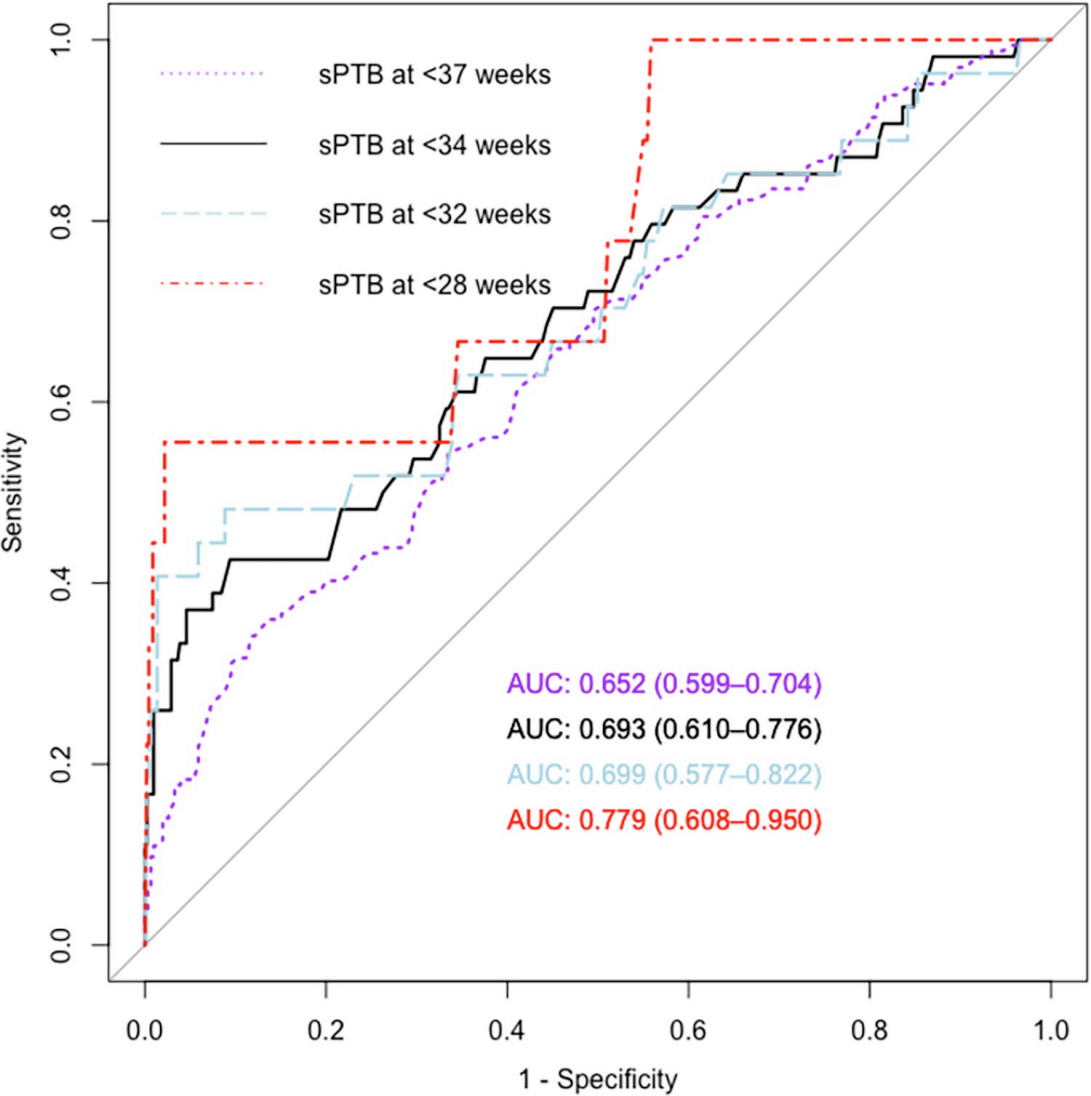
Receiver–operating characteristics (ROC) curve analysis showing the sensitivity and false-positive rate (1 − specificity) for spontaneous delivery before 28, 32, 34, 37 weeks at different cut-off levels of the cervical length during second trimester screening. sPTB–spontaneous preterm birth; AUC—area under the curve.

ROC curves showcased the moderate predictive value of cervical length screening, with the area under the curve (AUC) ranging from 0.65 for predicting sPTB at <37 weeks to AUC = 0.78 at <28 weeks (Fig 3). Various cut-off values were evaluated, including those derived by Youden’s index, the 5th centile, the 10th centile, F1-score, and an a-priori cut-off of 20 mm based on a meta-analysis [10]. Values for sensitivity, specificity, NPV, PPV, LR+, LR-, FPR, and FNR are also shown in Table 4.

**Table 4:** Cervical length screening performance for preterm birth prediction in twin pregnancy.

*Parameter for choosing cut-off*	*Cut-off*, *mm*	*Sensitivity*	*Specificity*	*PPV* ^*a*^	*NPV* ^*b*^	*Accuracy*	*LR+* ^*e*^	*LR-* ^*f*^	*FNR*^*g*^ *(%)*	*FPR*^*h*^ *(%)*
**Spontaneous preterm birth at <37 weeks (AUC**^**d**^ **0.65)**										
*5*^*th*^ *centile*	*25*.*1*	0.12	0.98	0.79	0.67	0.68	7.07	0.90	32.58	1.64
*10*^*th*^ *centile*	*30*.*4*	0.18	0.94	0.64	0.68	0.68	3.28	0.87	31.75	5.57
*F1-score = 0*.*54*	*40*.*2*	0.80	0.38	0.41	0.79	0.53	1.31	0.51	21.48	61.64
*Youden’s index = 0*.*22*	*32*.*8*	0.34	0.88	0.61	0.71	0.69	2.89	0.75	28.65	11.80
*A-priori*	*20*	0.09	0.99	0.88	0.67	0.68	13.02	0.92	33.11	0.66
**Spontaneous preterm birth at <34 weeks (AUC 0.69)**										
*5*^*th*^ *centile*	*25*.*1*	0.26	0.98	0.58	0.91	0.89	10.76	0.76	8.99	2.41
*10*^*th*^ *centile*	*30*.*4*	0.37	0.93	0.43	0.92	0.87	5.69	0.67	8.06	6.51
*F1-score = 0*.*43*	*28*.*5*	0.37	0.95	0.51	0.92	0.89	8.09	0.66	7.91	4.58
*Youden’s index = 0*.*33*	*31*.*2*	0.43	0.91	0.37	0.92	0.85	4.53	0.63	7.62	9.40
*A-priori*	*20*	0.22	0.99	0.75	0.91	0.90	23.06	0.79	9.27	0.96
**Spontaneous preterm birth at <32 weeks (AUC 0.70)**										
*5*^*th*^ *centile*	*25*.*1*	0.41	0.97	0.46	0.96	0.94	13.85	0.61	3.60	2.94
*10*^*th*^ *centile*	*30*.*4*	0.44	0.92	0.26	0.96	0.89	5.61	0.60	3.55	7.92
*F1-score = 0*.*5*	*20*.*5*	0.41	0.99	0.65	0.96	0.95	30.01	0.60	3.54	1.36
*Youden’s index = 0*.*39*	*20*.*5*	0.41	0.99	0.65	0.96	0.95	30.01	0.60	3.54	1.36
*A-priori*	*20*	0.37	0.99	0.63	0.96	0.95	27.28	0.64	3.75	1.36
**Spontaneous preterm birth at <28 weeks (AUC 0.78)**										
*5*^*th*^ *centile*	*25*.*1*	0.56	0.96	0.21	0.99	0.95	13.45	0.46	0.90	4.13
*10*^*th*^ *centile*	*30*.*4*	0.56	0.91	0.11	0.99	0.90	6.08	0.49	0.95	9.13
*F1-score = 0*.*47*	*10*.*4*	0.44	0.99	0.50	0.99	0.98	51.11	0.56	1.08	0.87
*Youden’s index = 0*.*53*	*19*	0.56	0.98	0.33	0.99	0.97	25.56	0.45	0.88	2.17
*A-priori*	*20*	0.56	0.98	0.31	0.99	0.97	23.23	0.46	0.88	2.39

^a^ Positive predictive value

^b^ Negative predictive value

^c^ Cervical length

^d^ Area under the curve

^e^ Positive likelihood ratio

^f^ Negative likelihood ratio

^g^ False negative rate

^h^ False positive rate.

To explore the potential improvement of the predictive value for the cut-offs outlined in Table 4, we conducted a comparative analysis involving different combinations of cervical length measurements along with chorionicity and funneling. These two characteristics were identified as independent predictors of sPTB at <34 weeks in Table 1. Building on the framework established in Table 4 and aiming to ensure comparability with previous studies [10, 15, 16], we selected cervical length cut-off values of 20 mm (approximating Youden’s index and F1-score cut-offs for 28 and 32 weeks), 25 mm (approximating the 5^th^ centile in our study), and 30 mm (approximating our 10^th^ centile). The findings are presented in Table 5.

**Table 5:** Cervical length measurement, funneling and chorionicity combined performance for spontaneous preterm birth prediction in twin pregnancy.

*Parameter/Cut-off*, *mm*	*Sensitivity*	*Specificity*	*PPV*	*NPV*	*Accuracy*	*+LR*	*-LR*	*FNR (%)*	*FPR (%)*
**Spontaneous preterm birth at <37 weeks**									
*Funneling*	0.07	1.00	0.92	0.67	0.67	20.46	0.94	33.48	0.33
*Presence of both funneling and cervical length below the cut-offs*									
*20*	0.05	1.00	0.90	0.66	0.67	16.74	0.95	33.77	0.33
*25*	0.06	1.00	0.91	0.66	0.67	18.60	0.94	33.62	0.33
*30*	0.07	1.00	0.92	0.67	0.67	20.46	0.94	33.48	0.33
*Presence of either funneling or cervical length below the cut-offs*									
*20*	0.10	0.99	0.89	0.67	0.68	14.88	0.91	32.82	0.66
*25*	0.12	0.99	0.83	0.67	0.68	8.83	0.90	32.51	1.31
*30*	0.18	0.96	0.70	0.69	0.69	4.29	0.85	31.46	4.26
*Presence of either funneling or cervical length below the cut-offs or monochorionic pregnancy*									
*20*	0.26	0.87	0.51	0.68	0.65	1.95	0.86	31.52	13.11
*25*	0.27	0.86	0.51	0.69	0.65	1.95	0.85	31.33	13.77
*30*	0.32	0.84	0.52	0.70	0.66	2.05	0.80	30.16	15.74
**Spontaneous preterm birth at <34 weeks**									
*Funneling*	0.19	1.00	0.83	0.90	0.90	38.43	0.82	9.63	0.48
*Presence of both funneling and cervical length below the cut-offs*									
*20*	0.15	1.00	0.80	0.90	0.90	30.74	0.86	10.02	0.48
*25*	0.17	1.00	0.82	0.90	0.90	34.58	0.84	9.83	0.48
*30*	0.19	1.00	0.83	0.90	0.90	38.43	0.82	9.63	0.48
*Presence of either funneling or cervical length below the cut-offs*									
*20*	0.26	0.99	0.78	0.91	0.91	26.90	0.75	8.87	0.96
*25*	0.28	0.98	0.65	0.91	0.90	14.41	0.74	8.74	1.93
*30*	0.37	0.94	0.47	0.92	0.88	6.68	0.67	7.98	5.54
*Presence of either funneling or cervical length below the cut-offs or monochorionic pregnancy*									
*20*	0.48	0.87	0.32	0.93	0.82	3.57	0.60	7.24	13.49
*25*	0.48	0.86	0.30	0.93	0.81	3.33	0.61	7.31	14.46
*30*	0.54	0.83	0.29	0.93	0.79	3.10	0.56	6.79	17.35
**Spontaneous preterm birth at <32 weeks**									
*Funneling*	0.26	0.99	0.58	0.96	0.95	22.92	0.75	4.38	1.13
*Presence of both funneling and cervical length below the cut-offs*									
*20*	0.26	0.99	0.70	0.96	0.95	38.20	0.75	4.36	0.68
*25*	0.26	0.99	0.64	0.96	0.95	28.65	0.75	4.37	0.90
*30*	0.26	0.99	0.58	0.96	0.95	22.92	0.75	4.38	1.13
*Presence of either funneling or cervical length below the cut-offs*									
*20*	0.37	0.98	0.56	0.96	0.95	20.46	0.64	3.77	1.81
*25*	0.41	0.97	0.48	0.96	0.94	15.01	0.61	3.59	2.71
*30*	0.44	0.93	0.28	0.96	0.90	6.34	0.60	3.52	7.01
*Presence of either funneling or cervical length below the cut-offs or monochorionic pregnancy*									
*20*	0.59	0.85	0.20	0.97	0.84	3.97	0.48	2.84	14.93
*25*	0.59	0.84	0.19	0.97	0.83	3.74	0.48	2.87	15.84
*30*	0.59	0.81	0.16	0.97	0.80	3.08	0.50	2.99	19.23
**Spontaneous preterm birth at <28 weeks**									
*Funneling*	0.33	0.98	0.25	0.99	0.97	17.04	0.68	1.31	1.96
*Presence of both funneling and cervical length below the cut-offs*									
*20*	0.33	0.98	0.30	0.99	0.97	21.90	0.68	1.31	1.52
*25*	0.33	0.98	0.27	0.99	0.97	19.17	0.68	1.31	1.74
*30*	0.33	0.98	0.25	0.99	0.97	17.04	0.68	1.31	1.96
*Presence of either funneling or cervical length below the cut-offs*									
*20*	0.56	0.97	0.28	0.99	0.96	19.66	0.46	0.89	2.83
*25*	0.56	0.96	0.22	0.99	0.95	14.20	0.46	0.90	3.91
*30*	0.56	0.92	0.12	0.99	0.91	6.73	0.48	0.94	8.26
*Presence of either funneling or cervical length below the cut-offs or monochorionic pregnancy*									
*20*	0.78	0.84	0.09	0.99	0.84	4.77	0.27	0.52	16.30
*25*	0.78	0.83	0.08	0.99	0.83	4.53	0.27	0.52	17.17
*30*	0.78	0.80	0.07	0.99	0.80	3.81	0.28	0.54	20.43

^a^ Positive predictive value

^b^ Negative predictive value

^c^ Preterm birth

^d^ Cervical length

^e^ Positive likelihood ratio

^f^ Negative likelihood ratio

^g^ false negative rate

^h^ False positive rate.

The results revealed that the adding extra factors to the screening process moderately impacted both false negative and false positive rates. When relatively loosened criteria (e.g., a positive result indicating either a short cervix, a monochorionic pregnancy, or funneling) were applied, only a slight improvement in the false negative rate was observed. However, this also led to a noticeable increase in the false positive rate compared to using cervical length as a sole criterion. Alternatively, by applying stricter criteria (e.g., a positive test meant both a of short cervical length and funneling were present), we saw a slight improvement in the false positive rates compared to using just cervical length as the sole criterion.

## Discussion

We retrospectively reviewed a cohort of 469 Taiwanese twin pregnancies. Short cervical length was significantly associated with spontaneous preterm deliveries, albeit with moderate predictive capability. Our ROC analysis revealed that cervical screening had the best predictive value for spontaneous preterm birth at <28 weeks. Cervical length in combination with other predictors, such as funneling and monochorionicity, provided additional details for decision-making. The major strength of the current study is its extended study period and a large cohort of Taiwanese twin pregnancies.

Similar to a large meta-analysis, our study confirmed that cervical length screening had the highest AUC for predicting spontaneous preterm birth at <28 weeks, with a decrease in predictive accuracy observed at higher gestational age at birth [10]. Being in line with those reported in other studies [16, 23, 32], our AUC values were moderate, suggesting that although cervical length screening is helpful in predicting spontaneous preterm birth, there are still some limitations to be addressed. Most importantly, the screening test was more effective at identifying high-risk cases rather than ruling out lower-risk ones. Although some authors underlined the ability of cervical length screening to identify lower-risk cases due to high negative predictive value [14], our results illustrate the limited ability of cervical length screening to rule out sPTB in twins. High negative likelihood ratios indicate that a cervical length measurement above the cut-off does not significantly change the pretest probability, thereby not sufficient to exclude the subsequent development of sPTB.

Conversely, we could identify a very high-risk group for sPTB by utilizing cervical length screening, as shown in Fig 2, those women with cervical length measurements below 10 mm had a rate of sPTB at <34 weeks as high as 75%. Timely identification of high-risk group allows for earlier intervention and more preventative pregnancy management. To date, the literature regarding preventive strategies is inconclusive, although there are studies supporting universal cervical length mid-pregnancy screening and consequent vaginal progesterone therapy for high-risk twin pregnancies [33, 34], as well as cerclage placement in those with cervical length shorter than 15 mm [35, 36].

Besides, our study suggested that a cervical length cut-off value of 20 mm had the highest LR+ for prediction of sPTB at all gestational ages at birth, except for <28 weeks, where the cut-off of 10.4 mm demonstrated the highest LR+. These findings align with a meta-analysis that also supported a cut-off point at 20 mm [10]. We further explored the predictive value of adding funneling and chorionicity to the screening process. Our results were consistent with previous studies, indicating that funneling is a significant predictor of sPTB in twin pregnancies [15, 37, 38]. Additionally, the results were in line with those studies suggesting that monochorionicity is significantly associated with spontaneous preterm birth [39].

Current cervical length screening protocol, however, demonstrated only moderate predictive value even when taking into consideration funneling and monochorionicity, as evident from the results presented in Table 5. This observation suggests the existence of unknown pathogenetic mechanisms specific to multiple pregnancies in the spontaneous onset of preterm birth [40, 41]. Further research into the mechanisms of sPTB in twins is warranted. Understanding the pathogenesis could inspire the development of new screening tests for sPTB in twins with enhanced predictive value. Such improvement is essential for better allocation of healthcare resources, potentially reducing unnecessary interventions and stress for expectant mothers [14, 42, 43], while focusing on higher-risk cases to shorten the length of hospital stays and prevent severe adverse outcomes. The likelihood ratios provided in this study might support the integration of Bayesian reasoning into clinical practice and medical education [27, 28].

There are some important limitations of this study. First, its retrospective nature implies that data were collected for purposes other than research. As a result, we were unable to ascertain variables and outcomes such as shape and degree of funneling, the exact dosage and methods of administering progesterone, tocolytics, information about hospitalization, and surgical techniques for cerclage placement. These factors should be considered in future prospective cervical length and spontaneous preterm birth risk research. Second, a high rate of cesarean section (~90%) was reported in our sample, but it is consistent with a trend for multiple pregnancies in East Asian countries with similar economies [44–48]. Third, the analysis included pregnancies with potential confounding factors. Often those cases who underwent cerclage, progesterone, or tocolytic treatment are excluded from the analysis, even though they represent the pregnancies with the highest risk for sPTB. The logistic regression analysis adjusting for interventions, maternal, and pregnancy characteristics suggests that despite the effect of those confounders on the correlation between cervical length and sPTB, cervical length remained a significant predictor for spontaneous preterm birth in twin pregnancies. We thus decided to include these potentially confounding cases in our sample. Although this method is imperfect, those confounders might have reduced the predictive value of cervical length. To further account for multiple interventions, we provided a table comparing groups that received cerclage, progesterone, or tocolytics as a supporting material (S1 Table), those receiving cervical cerclage (n = 7) had significantly shorter cervical length and more frequently had funneling compared to the total sample. As for the progesterone group, a significantly higher rate of IVF pregnancies compared to the total sample should be noted (p-value<0.001). It can be a result of the fact, that often progesterone is administered as a part of IVF protocol. On the other hand, both multivariable regression and S1 Table point out that tocolysis was associated with an earlier gestational age of delivery. Frequently, tocolytic agents are administered in twin pregnancies with signs of early onset of labor and the main purpose is to complete a course of corticosteroids for lung maturation, not to stop preterm labor [49]. And lastly, our clinic is a private center in Taipei City, which might affect the generalizability of our findings. However, twin pregnancies are often resulting from artificial reproductive technologies, and being high-risk, they tend to be referred or seek care outside of public health care settings. Therefore, our sample might not differ from the general population of twin pregnancies in Taiwan.

In conclusion, our results suggest that the cervical length distribution was similar to that of Caucasian twin pregnancy studies, and had a greater negative skew compared to cervical length distribution in Taiwanese singletons. We provide likelihood ratios as a tool for improving decision-making and pregnancy management. While our data enables clinicians to choose optimal screening parameters based on their goals and the prevalence of preterm birth in the specific population, we found that cut-off at 20 mm showed a reasonable balance between false negatives and positives in predicting spontaneous preterm birth. Adequate risk identification allows for appropriate pregnancy management, better allocation of healthcare resources, and a reduction in unnecessary stress for falsely identified high-risk cases.

## Supporting information

S1 Table Comparison between groups received intervention. The table containing stratified analysis by intervention (cerclage, progesterone, tocolysis).
(XLSX)

S2 Table Anonymized data. (XLSX)
